# Sex and menopause impact ^31^P-Magnetic Resonance Spectroscopy brain mitochondrial function in association with ^11^C-PiB PET amyloid-beta load

**DOI:** 10.1038/s41598-022-26573-5

**Published:** 2022-12-21

**Authors:** Steven Jett, Jonathan P. Dyke, Caroline Andy, Eva Schelbaum, Grace Jang, Camila Boneu Yepez, Silky Pahlajani, Ivan Diaz, Roberta Diaz Brinton, Lisa Mosconi

**Affiliations:** 1 Department of Neurology, Weill Cornell Medicine, New York, NY 10021 USA; 2 Department of Radiology, Weill Cornell Medicine, New York, NY USA; 3 Department of Population Health Sciences, Weill Cornell Medicine, New York, NY USA; 4 Department of Pharmacology, University of Arizona, Tucson, AZ USA; 5 Department of Neurology, University of Arizona, Tucson, AZ USA

**Keywords:** Cognitive ageing, Predictive markers, Risk factors, Neuroscience, Alzheimer's disease, Magnetic resonance imaging, Positron-emission tomography, Hormones, Mitochondria, Menopause

## Abstract

Increasing evidence implicates sex and endocrine aging effects on brain bioenergetic aging in the greater lifetime risk of Alzheimer’s disease (AD) in women. We conducted ^31^Phosphorus Magnetic Resonance Spectroscopy (^31^P-MRS) to assess the impact of sex and menopause on brain high-energy phosphates [adenosine triphosphate (ATP), phosphocreatine (PCr), inorganic phosphate (Pi)] and membrane phospholipids [phosphomonoesters/phosphodiesters (PME/PDE)] in 216 midlife cognitively normal individuals at risk for AD, 80% female. Ninety-seven participants completed amyloid-beta (Aβ) ^11^C-PiB PET. Women exhibited higher ATP utilization than men in AD-vulnerable frontal, posterior cingulate, fusiform, medial and lateral temporal regions (p < 0.001). This profile was evident in frontal cortex at the pre-menopausal and peri-menopausal stage and extended to the other regions at the post-menopausal stage (p = 0.001). Results were significant after multi-variable adjustment for age, APOE-4 status, midlife health indicators, history of hysterectomy/oophorectomy, use of menopause hormonal therapy, and total intracranial volume. While associations between ATP/PCr and Aβ load were not significant, individuals with the highest Aβ load were post-menopausal and peri-menopausal women with ATP/PCr ratios in the higher end of the distribution. No differences in Pi/PCr, Pi/ATP or PME/PDE were detected. Outcomes are consistent with dynamic bioenergetic brain adaptations that are associated with female sex and endocrine aging.

## Introduction

The prevalence of late-onset Alzheimer’s disease (AD) is greater in women than in men^1^, independent of age and survival rates^2–4^. Currently, post-menopausal women account for over 60% of all AD cases^1^. Moreover, female carriers of the Apolipoprotein E epsilon 4 (APOE-4) risk allele are affected earlier and in higher numbers than male carriers, though both sexes experience elevated risk relative to non-carriers^5,6^.

Female sex is inextricably linked to the midlife neuro-endocrine aging transition of the menopause^7^. Mounting evidence from preclinical and translational studies identifies deprivation of estrogenic regulation of brain bioenergetics and neuroprotective effects following menopause as key biological mechanisms underlying women’s greater lifetime risk of AD^2,3^. Further, AD pathology begins during a ~ 20 year pre-symptomatic phase^8^, thus proximate to the menopause transition, further highlighting links between midlife endocrine aging and AD risk in women^7^.

Preclinical research indicates that menopause impacts multiple neurobiological systems, brain bioenergetics in particular^9–14^. Under normal conditions, the brain utilizes glucose as its primary fuel source, a process that is dependent on 17β-estradiol^7^. In animal studies, estrogenic regulation of metabolic pathways falters during perimenopause, triggering declines in cerebral glucose metabolism (CMRglc) paralleled by increases in mitochondrial utilization of ketone bodies, lipids and amino acids as alternative fuels for adenosine triphosphate (ATP) production^15–18^. Over time, however, this prompts mitochondrial dysfunction, amyloid-beta (Aβ) dysmetabolism^19^ and cellular apoptosis in female animals^16^. Brain glucose hypometabolism, mitochondrial dysfunction, and reduced oxidative phosphorylation (OXPHOS) are consistent findings in AD^20–22^, and may precede formation of AD plaques^23^.

In translational neuroimaging studies, peri-menopausal and post-menopausal women exhibit reduced CMRglc on ^18^F-fluoro-deoxygluose (FDG) PET and higher Aβ deposition on ^11^C-Pittsburgh Compound B (PiB) PET, a hallmark of AD pathology, as compared to pre-menopausal women and to age-controlled men^9–14^. The extent of glucose hypometabolism exceeded that of Aβ load^10^, further identifying midlife hypometabolism as an early female-specific indicator of prodromal AD^7^.

Because the FDG-PET signal is based on trapping fluoro-deoxygluose after it is phosphorylated into deoxy-glucose-6-phosphate (the first step in glycolysis), the technique does not provide direct information on mitochondria OXPHOS or ATP production. ^31^Phosphorus Magnetic Resonance Spectroscopy (^31^P-MRS) is the only neuroimaging technique currently available that enables in vivo assessment of cerebral mitochondrial function through the detection of intracellular high-energy phosphates (HEP) such as ATP, phosphocreatine (PCr), and inorganic phosphate (Pi)^24–26^. The chemical exchange of phosphate moieties between PCr ⇄ ATP ⇄ Pi is key to maintaining a stable cellular ATP concentration by ensuring continuous energy supply for electrophysiological activity and cerebral bioenergetics^20^.

The first generation of ^31^P-MRS studies performed at 1.5 Tesla with only surface coil localization and limited brain coverage reported mixed findings of increased^27,28^ or unchanged^29^ HEP metabolite levels in AD. More recent studies using whole-brain, multi-slice ^31^P-MRS have reported alterations in HEP metabolites in AD as well as Mild Cognitive Impairment (MCI) as compared to healthy controls^30–32^, which have been interpreted as dysregulation of neuroenergetic pathways^33^. Changes in ^31^P-MRS-derived phosphomonoesters (PME) and phosphodiesters (PDE) composition have also been noted in AD^34,35^. It is unknown whether HEP or phospholipid metabolites are altered in asymptomatic individuals *at risk* for AD, and whether they are associated with Aβ deposition in midlife, when potential for preservation of cognitive function and AD prevention is greatest.

Herein, we conducted a whole-brain, multi-slice ^31^P-MRS study to test for effects of sex and menopausal status on HEP metabolites and membrane phospholipids among over 200 cognitively normal midlife men and women carrying risk factors for AD (e.g. family history of late-onset AD and/or APOE-4 genotype). We also examined associations between phosphorus metabolites and fibrillar Aβ load as measured with ^11^C-PiB PET scans.

## Results

### Participants

We enrolled 230 participants for this study. Of these, 14 were excluded due to incidental findings on MRI (n = 5 small vessel disease or lacunar infarctions, n = 2 meningiomas, n = 1 mild hydrocephalus, n = 1 demyelination), MR artifacts (n = 2), or incomplete ^31^P-MRS studies (n = 3). The remaining 216 participants were examined in this study, including 170 women and 46 men with complete clinical exams, menopause status assessments, and ^31^P-MRS exams. Ninety-seven (45%) participants completed ^11^C-PiB PET exams.

Participant characteristics are shown in Table 1. There were no differences for demographic and clinical measures between men and women. The female group included 39 pre-menopausal, 61 peri-menopausal, and 70 post-menopausal women. On post-hoc examination of menopause status, the male group and the post-menopausal group included more cases with hypertension as compared to the pre-menopausal group (p = 0.05). Hypertension was included as a confounder in all analyses, as described in “Methods”.

**Table 1:** Participant characteristics.

	Women			Men
	Pre-menopausal	Peri-menopausal	Post-menopausal	
N	39	61	70	46
Age, mean, range, years	44, 40–50	49, 40–58	55, 41–65	51, 40–65
Education, years	17(2)	17(2)	17(2)	18(2)
Race, % White	74	72	82	72
APOE-4 status, % positive	51	34	44	52
AD family history, % positive	56	72	59	61
Smoking, % current or past	29	21	21	11
Hypertension, % positive	0	8	10*	17*
Hypercholesterolemia, % positive	30	28	40	24
Hyperinsulinemia, % positive	23	38	29	31
Hysterectomy or oophorectomy, % positive	0	2	20	n.a
MHT use, % users	0	20	40	n.a

Measures are mean (SD) unless otherwise specified.

*Different from pre-menopausal group, p < 0.05.

Global cognition and memory scores did not differ by sex or menopausal status (Table 2).

**Table 2:** Sex and menopause effects on cognition.

Exposure	Outcome	Group	Mean	Std. error	95% C.I		P
Sex	Global cognition	Women	− 0.012	0.089	− 0.187	0.163	0.648
		Men	0.034	0.045	− 0.056	0.123	
	Memory	Women	0.031	0.115	− 0.197	0.258	0.921
		Men	0.018	0.059	− 0.099	0.134	
Menopause status	Global cognition	Pre-menopause	− 0.037	0.109	− 0.252	0.178	0.402
		Peri-menopause	− 0.049	0.078	− 0.202	0.104	
		Post-menopause	0.145	0.084	− 0.019	0.310	
		Men	− 0.008	0.089	− 0.183	0.166	
	Memory	Pre-menopause	− 0.040	0.143	− 0.321	0.241	0.970
		Peri-menopause	0.009	0.101	− 0.191	0.209	
		Post-menopause	0.058	0.109	− 0.157	0.274	
		Men	0.032	0.116	− 0.196	0.260	

Multi-variable adjusted, standardized mean (SE) and 95% confidence intervals (CI).

### Sex differences in phosphorus metabolites

**Figure 1:**
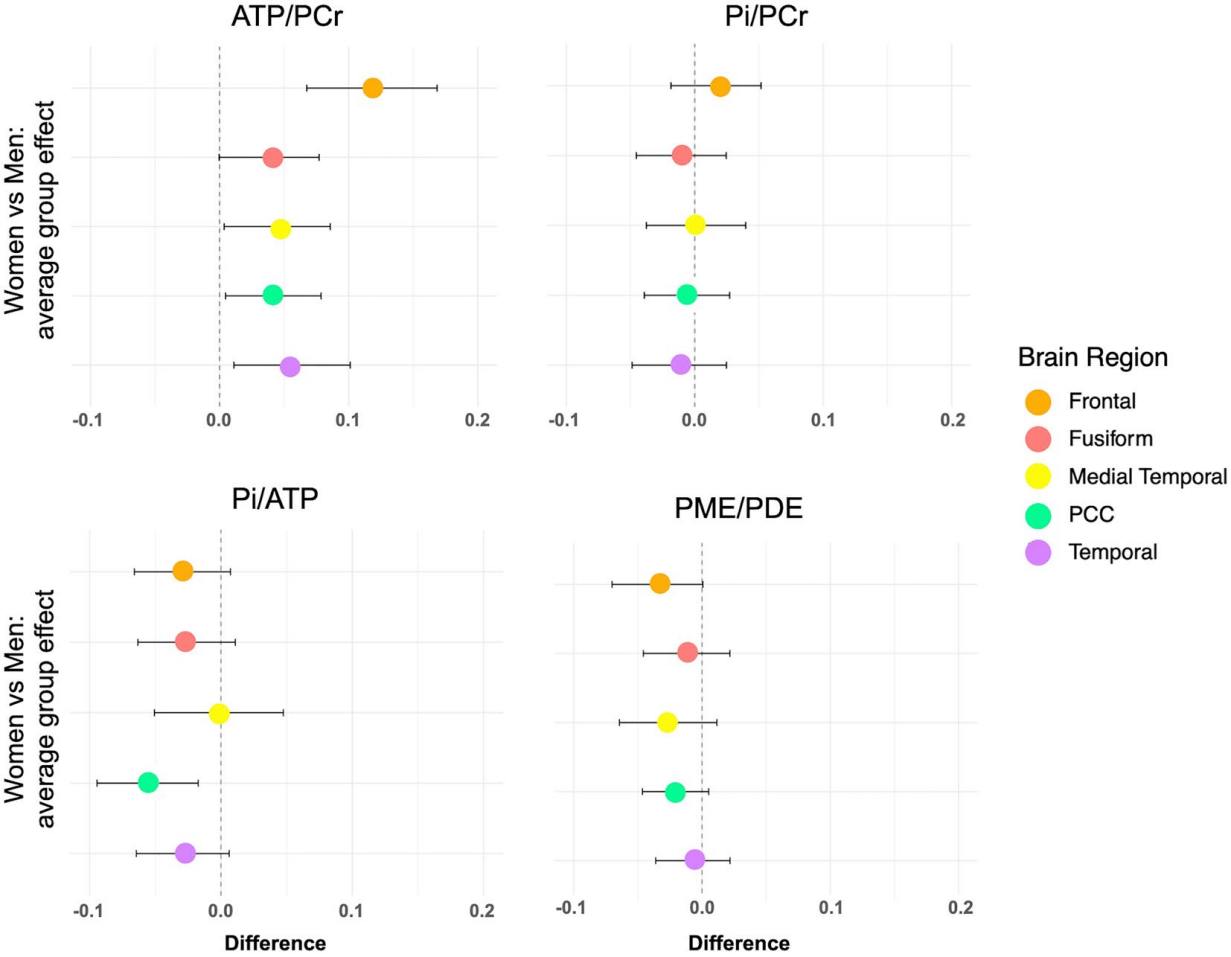
Effects of sex on HEP and membrane phospholipids. Forest plots constructed from linear regression models for each brain region, outcome and pairwise comparisons for groups. The value on the x axis is the estimated average causal effect of exposure (female sex) compared to the reference (male sex), with 95% confidence intervals (C.I.). Values to the right of the vertical line show higher values for women compared to men. ATP, total adenosine triphosphate; PCr, phosphocreatine; Pi, inorganic phosphate; PDE, phosphodiesters; PME, phosphomonoesters.

Adjusting by age, APOE-4 status and total intracranial volume, main effects of sex were observed for ATP/PCr, a marker of ATP utilization^12^ (p < 0.001). As compared to men, women exhibited higher ATP/PCr levels in all regions examined (p = 0.002; Table 3). These effects remained significant after adjustment for midlife health indicators and menopause-related factors, with the largest effect size in frontal cortex (Fig. 1).

**Table 3:** Sex differences in regional phosphorus metabolite levels.

Metabolite	Region	Group	Mean	Std. error	95% C.I		P*
ATP/PCr	Frontal	Female	0.410	0.010	0.390	0.429	0.002
		Male	0.351	0.010	0.331	0.370	
	Fusiform	Female	0.916	0.016	0.883	0.948	
		Male	0.880	0.016	0.848	0.912	
	Medial temporal	Female	0.242	0.004	0.233	0.250	
		Male	0.231	0.004	0.223	0.239	
	PCC	Female	0.366	0.009	0.349	0.384	
		Male	0.344	0.009	0.327	0.362	
	Lateral temporal	Female	0.322	0.005	0.312	0.333	
		Male	0.306	0.005	0.296	0.317	1.000
Pi/ATP	Frontal	Female	0.490	0.031	0.430	0.551	
		Male	0.539	0.030	0.479	0.599	
	Fusiform	Female	0.198	0.012	0.174	0.221	
		Male	0.217	0.012	0.194	0.241	
	Medial temporal	Female	6.928	0.218	6.500	7.358	
		Male	6.978	0.216	6.552	7.404	
	PCC	Female	0.471	0.021	0.430	0.514	
		Male	0.537	0.021	0.495	0.578	
	Lateral temporal	Female	0.541	0.036	0.469	0.613	
		Male	0.610	0.036	0.539	0.681	1.000
Pi/PCr	Frontal	Female	0.098	0.005	0.088	0.107	
		Male	0.091	0.005	0.081	0.100	
	Fusiform	Female	0.179	0.009	0.162	0.196	
		Male	0.186	0.008	0.169	0.202	
	Medial temporal	Female	0.044	0.002	0.041	0.048	
		Male	0.044	0.002	0.040	0.048	
	PCC	Female	0.085	0.005	0.075	0.096	
		Male	0.087	0.005	0.077	0.097	
	Lateral temporal	Female	0.058	0.003	0.052	0.064	
		Male	0.060	0.003	0.054	0.066	
PME/PDE	Frontal	Female	1.659	0.114	1.434	1.884	0.688
		Male	1.903	0.113	1.679	2.126	
	Fusiform	Female	0.919	0.046	0.829	1.009	
		Male	0.956	0.045	0.866	1.045	
	Medial temporal	Female	4.272	0.280	3.720	4.824	
		Male	4.699	0.278	4.151	5.247	
	PCC	Female	1.684	0.204	1.281	2.087	
		Male	2.071	0.203	1.671	2.471	
	Lateral temporal	Female	3.127	0.240	2.653	3.600	
		Male	3.280	0.238	2.810	3.750	

Multi-variable adjusted, unstandardized mean (SE) and 95% confidence intervals (CI) by group (men, women). *Bonferroni corrected P values.

ATP, total adenosine triphosphate; PCC, posterior cingulate cortex and precuneus; PCr, phosphocreatine; Pi, inorganic phosphate; PDE, phosphodiesters; PME, phosphomonoesters.

There were no main effects of sex on Pi/PCr (e.g., energy demand^36^), Pi/ATP (e.g., ATP hydrolysis^37^) or PME/PDE ratios (e.g. phospholipid turnover rate^38^) (Table 3). Descriptively, women exhibited lower Pi/ATP in posterior cingulate (PCC) as compared to men, which did not survive correction for multiple comparisons (Fig. 1).

### Effects of menopause status

**Figure 2:**
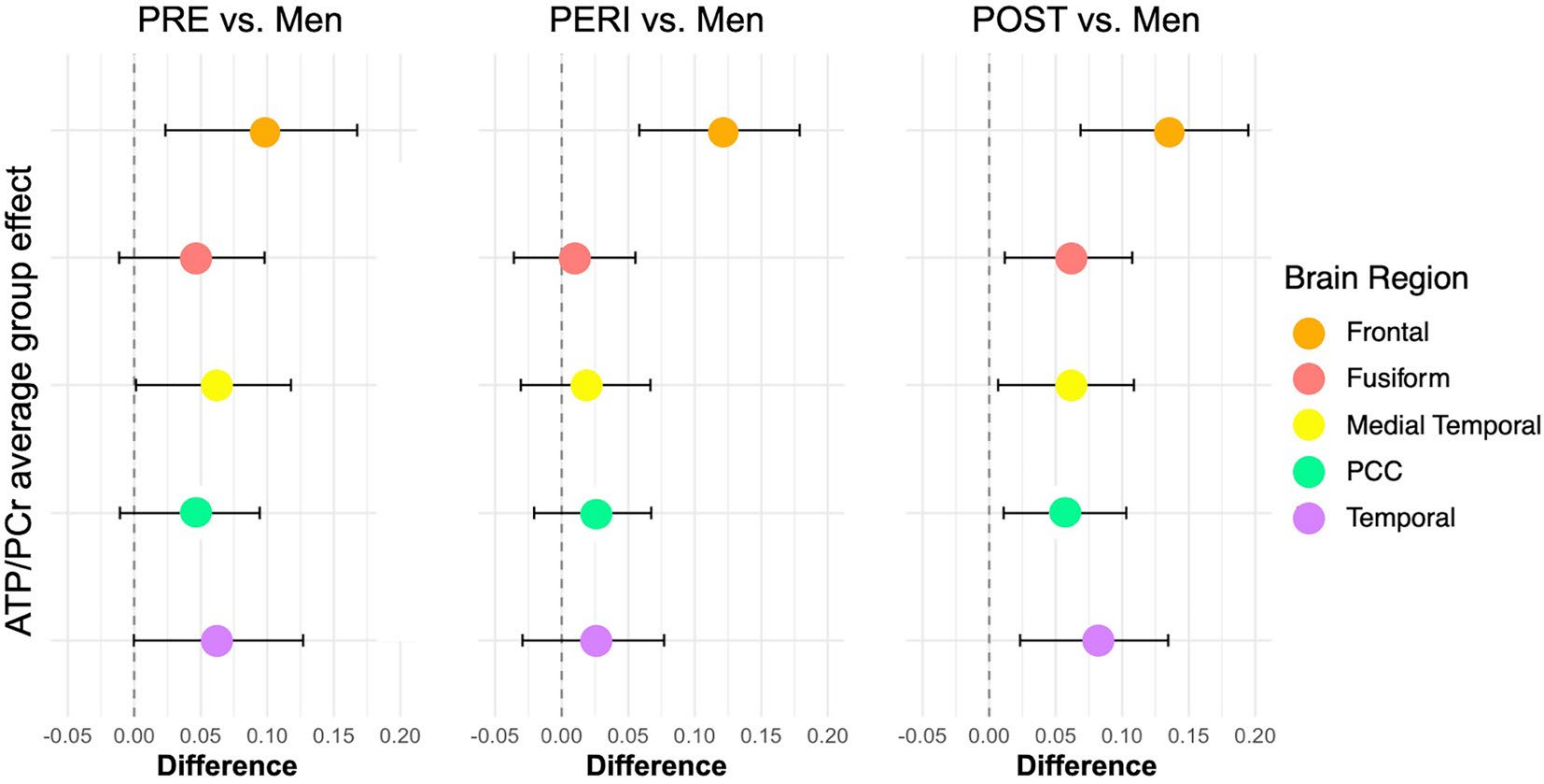
Effects of menopause status on ATP/PCr measures. Forest plots constructed from linear regression models for each brain region, outcome and pairwise comparisons for groups. The value on the x axis is the estimated average causal effect of exposure (each menopausal group) compared to reference (men), with 95% confidence intervals (C.I.). Values to the right of the vertical line show higher values for each menopausal group compared to men. ATP, total adenosine triphosphate; PCr, phosphocreatine; PERI, peri-menopause; POST, post-menopause; PRE, pre-menopause.

Menopause status effects were observed for ATP/PCr measures (p < 0.001) but not for the other measures, although a trend was noted in temporal cortex (p = 0.064). On pair-wise post-hoc analysis, the post-menopausal group exhibited higher ATP/PCr relative to men across all regions examined (p < 0.001). These effects remained significant after adjustment for midlife health indicators and menopause-related factors, with the largest effect size in frontal cortex (Fig. 2). The pre-menopausal and peri-menopausal groups also exhibited higher ATP/PCr in frontal cortex relative to men (p < 0.05), which was not observed in other regions (Table 4, Fig. 2).

**Table 4:** Menopause effects on regional ATP utilization.

Region	Group	Mean	Std. error	95% C.I		P*
Frontal	Pre-menopause	0.402	0.013	0.377	0.427	< 0.001
	Peri-menopause	0.409	0.009	0.391	0.427	
	Post-menopause	0.414	0.009	0.396	0.432	
	Male	0.351	0.010	0.331	0.370	
Fusiform	Pre-menopause	0.920	0.021	0.879	0.961	0.242
	Peri-menopause	0.891	0.015	0.861	0.920	
	Post-menopause	0.933	0.015	0.903	0.963	
	Male	0.880	0.016	0.848	0.912	
Medial temporal	Pre-menopause	0.244	0.005	0.233	0.254	0.199
	Peri-menopause	0.236	0.004	0.229	0.244	
	Post-menopause	0.245	0.004	0.237	0.252	
	Male	0.231	0.004	0.223	0.239	
PCC	Pre-menopause	0.371	0.011	0.349	0.393	0.541
	Peri-menopause	0.359	0.008	0.343	0.375	
	Post-menopause	0.370	0.008	0.354	0.386	
	Male	0.344	0.009	0.327	0.362	
Lateral temporal	Pre-menopause	0.323	0.007	0.310	0.337	0.064
	Peri-menopause	0.314	0.005	0.305	0.324	
	Post-menopause	0.329	0.005	0.319	0.339	
	Male	0.306	0.005	0.296	0.317	

Multi-variable adjusted, unstandardized mean (SE) and 95% confidence intervals (CI) by sex and menopause status. *Bonferroni corrected P values.

ATP, total adenosine triphosphate; PCC, posterior cingulate cortex and precuneus; PCr, phosphocreatine.

### Associations between phosphorus metabolites and cognition

There were no significant associations between frontal ATP/PCr and global cognition or verbal memory scores in women (p = 0.717 and p = 0.246, respectively) or men (p = 0.495 and p = 0.151, respectively). Given lack of associations between frontal ATP/PCr and cognition scores, and lack of menopause status effects on cognition, testing of associations by menopause status was not conducted.

### Associations between phosphorus metabolites and amyloid-beta load

**Figure 3:**
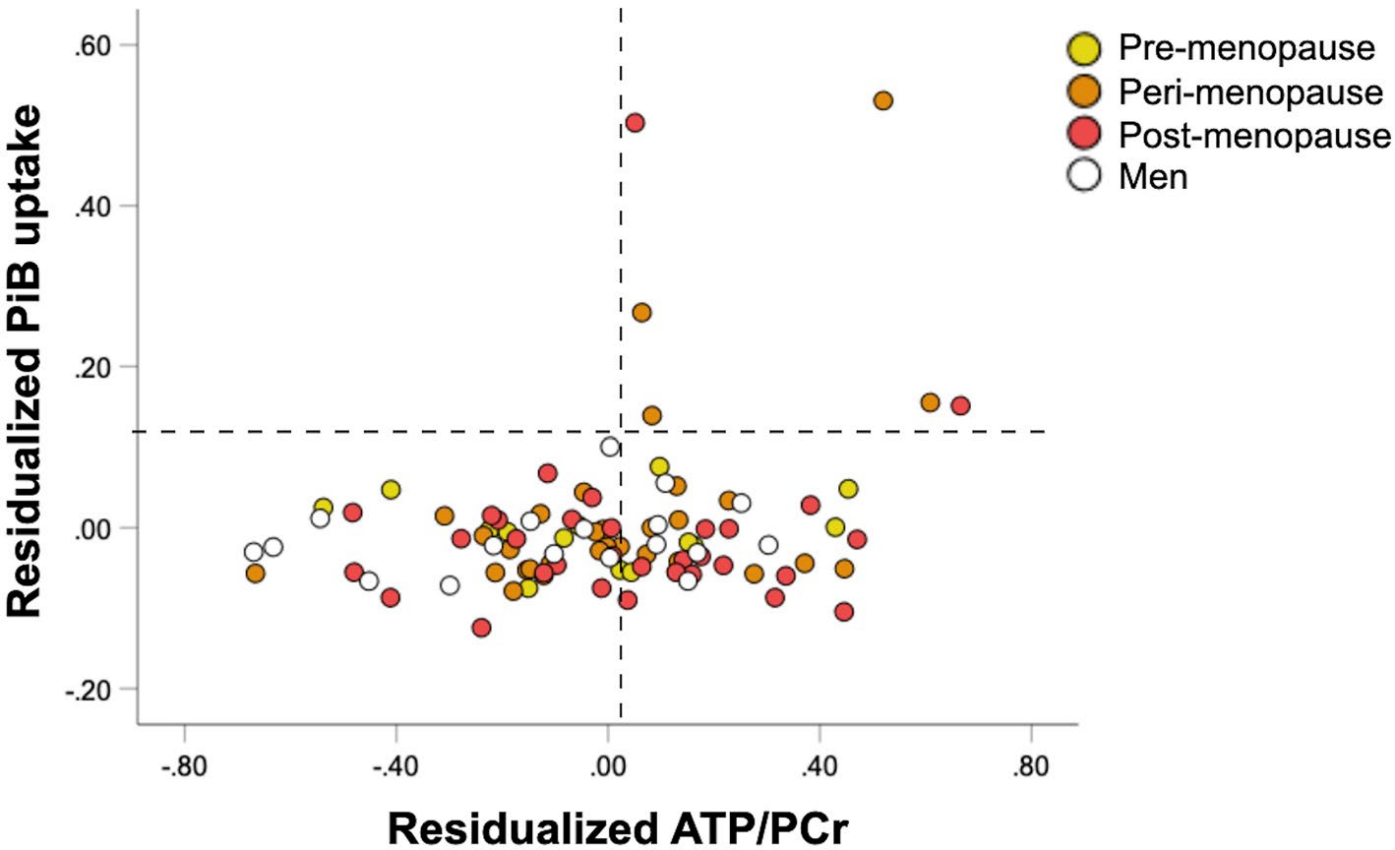
Associations of ATP/PCr and amyloid-beta load. Scatterplots showing associations between ATP/PCr in frontal cortex and PiB SUVR in AD-mask, in men (white) and women (grey). ATP/PCr measures are residualized by age, APOE-4 status and total intracranial volume. PiB SUVR measures are residualized by age and APOE-4 status.

Among the subset of 97 participants with ^11^C-PiB PET exams, there were no significant associations between regional ATP/PCr and PiB uptake (p = 0.989). However, the six participants exhibiting the highest PiB SUVR also exhibited ATP/PCr ratios towards the higher end of the distribution (Fig. 3). These participants were postmenopausal (n = 3) or perimenopausal women (n = 3), all with a family history of AD, and 67% were APOE-4 carriers.

## Discussion

In this ^31^P-MRS study of cognitively normal midlife individuals at risk for AD, women exhibited higher brain ATP re-synthesis (e.g. ATP utilization) in brain regions vulnerable to AD as compared to age-controlled men. These effects were more widespread in the post-menopausal group, and were independent of age, APOE-4 status, total intracranial volume, midlife health indicators, menopause type (surgical vs. spontaneous), and history of HT use. Although associations between ATP utilization and PiB uptake were not significant, the participants with the highest Aβ burden were post-menopausal and peri-menopausal women with ATP/PCr levels in the higher end of the distribution.

There is increasing evidence that AD prevalence, symptomatology, and pathophysiology vary by sex^2–4^. Importantly, women sustain clinically-defined normal memory performance for longer than men^39^ in spite of experiencing an earlier onset^9–14^ and accelerated progression of AD pathology^40–43^. It has been suggested that sex differences in cerebral metabolism might explain the divergent trajectories, possibly offsetting onset of clinical symptoms in women^44^.

Evidence for sex differences in brain ATP utilization is limited. Currently, only two ^31^P-MRS studies have addressed this question. One study of the adult lifespan reported higher ATP utilization in frontal, temporal, and occipital cortices of women ages 20–85 years as compared to age-controlled men^36^. The other study also reported higher ATP utilization in frontal and temporal regions of post-menopausal women as compared to age-controlled men, controlling for age and APOE-4 status^12^. As neither study focused specifically on individuals at risk for AD, or tested for associations between phosphorus metabolites and AD biomarkers, it is unclear whether ATP differences were related to AD.

^31^P-MRS investigations of HEP metabolites in AD are limited by small sample sizes, methodological differences, as well as differences in reported metabolite outcomes and brain regions examined. Overall, older studies comparing generally small samples of AD patients and elderly controls reported contrasting results of elevated PCr^27^ or ATP^28^, or no effects^29,45^. Such discrepancies are likely due to the fact that these studies were performed at 1.5 Tesla with only surface coil localization and lower SNR than 3.0 Tesla, which limited coverage and introduced inhomogeneous spin excitation^46^. Modern systems operating at 3 Tesla or higher, with volume or phased array coils, have allowed for improved ^31^P-MRS coverage and sensitivity, which provides the opportunity to explore regional differences in the brain. A recent multi-slice, whole-brain ^31^P-MRS study of 11 AD, 15 amnestic MCI, and 15 controls reported that as compared to age-matched controls, both AD and amnestic MCI patients exhibited higher ATP utilization^30^. Another recent study reported higher PCr/Pi levels in a sample of 31 AD patients compared to 31 controls, but no differences in γ- or β-ATP concentrations^31^. However, elevated γ-ATP concentrations in AD relative to controls have been reported by others^28,32^. Overall, while more studies are needed to clarify HEP dynamics in AD, most studies point to increased ATP utilization^33^, possibly reflecting metabolic stress in AD brain.

The present study, with a sample of 216 individuals carrying established risk factors for AD, also shows higher ATP utilization in frontal and lateral temporal cortices of middle-aged women as compared to age-controlled men. Further, findings reported herein provide novel evidence that greater ATP utilization also involves PCC, fusiform, and medial temporal lobes, including hippocampus, amygdala, parahippocampal and entorhinal cortex. Additionally, sex differences in ATP utilization were influenced by menopause status. Both pre-menopausal and peri-menopausal groups exhibited higher ATP/PCr than men in frontal cortex, whereas the post-menopausal group exhibited higher ATP/PCr in additional regions with known vulnerability to early pathological and metabolic changes in AD, and higher specificity for AD than neocortical areas^47,48^. As the subset of individuals exhibiting the highest Aβ burden were all peri-menopausal and post-menopausal women with ATP/PCr levels towards the higher end of the distribution, this further suggests female-specific AD-related effects on brain energy metabolism with onset in peri-menopause.

Under normal aerobic conditions, an increase in energy demand is matched by a corresponding increase in mitochondrial ATP production, the latter being reflected by a proportional increase in the ATP/PCr ratio^20^. Therefore, our data suggest that more effort is required to maintain stable energy production in women’s frontal cortices than in men’s, and that the effort increases in AD-related brain regions at the post-menopausal stage. It also indicates that the menopause transition is accompanied by a redistribution in the content of metabolites involved in the creatine kinase equilibrium, leading to widespread higher ATP utilization in frontal cortex after the cessation of ovarian 17β-estradiol (estrogen) production.

A burgeoning array of studies has provided evidence for neuroprotective effects of estrogen, and identified life-time estrogen exposure as a modulator of cognitive aging in women^4,7,49^. Preclinical studies demonstrate that estradiol withdrawal during the menopause transition triggers the disassembly of the systems required for cerebral glucose utilization and ketogenic pathway suppression^15,17^. This in turn triggers compensatory mechanisms involving increased breakdown of amino acids, fatty acids (β-oxidation), and ketone bodies in mitochondria to preserve ATP production^15–18^. Continued dependence on these pathways prompts white matter catabolism^18^, Aβ dysmetabolism^19^, and neuronal degeneration in female animals^16,49^. In mouse models of AD, decreasing estradiol levels following oophorectomy exacerbate brain damage under neurodegenerative conditions^50^ and trigger increases in Aβ fibrillization^51^. Notably, estrogen-inducible neuroprotective mechanisms converge onto mitochondria, which are pivotal to sustaining calcium homeostasis and cell survival^52^, and are a site of estradiol synthesis^53^, further highlighting their involvement in sex- and endocrine aging-related effects on AD risk.

Brain imaging studies also identify the menopause transition as a driver of the timing and progression of AD^3,4,7^. Peri-menopausal and post-menopausal women exhibit progressively lower CMRglc, higher Aβ load, and lower gray and white matter volume as compared to pre-menopausal women and age-controlled men^9–14^. In light of previous findings, present results of higher brain ATP utilization might reflect a compensatory reaction to previously reported hypoglycolytic metabolism occurring during the menopause transition^9–13^. These findings are supported by mechanistic analyses that demonstrate a shift from brain glucose to lipid metabolism across midlife endocrine aging, resulting in catabolism of white matter as a source of lipid for generation of ketone bodies^54^ and restoration of ATP^15–18^. The loss of estrogenic controlled glucose metabolism results in a 20–25% decline in CMRglc and mitochondrial respiratory efficiency followed by a compensatory bioenergetic adaptive response to utilize lipids as an auxiliary fuel^7,16,18,55^. The utilization of high energy lipid fuel is therefore consistent with the rise in ATP generation in select brain regions and consistent with decline in white matter integrity and volume observed in preclinical female endocrine aging models^7,16,18,54,55^, a shift from efficient to uncoupled mitochondrial respiration^16,54,55^, and the loss of white matter volume in midlife women^10,12^. In this study, there were no significant sex differences in PME/PDE ratios after correction for multiple comparisons. However, descriptively, women showed lower PME/PDE than men in frontal cortex (Table 3 and Fig. 1), which may be related to the increase in fat catabolism, or higher phospholipid turnover observed in animal models. PME are membrane precursors that play an important role in the synthesis of membrane lipids, whereas PDE are the products of phospholipid breakdown. Some ^31^P-MRS studies have reported PME/PDE alterations in AD^34,35,38,56^, while others show no differences^30,31,33,45^. Given lack of main effects of sex on PME/PDE, we did not further test for menopause status effects. More work is needed to test for associations between ATP utilization and PME/PDE turnover as a function of menopause status, and to examine whether sex differences in phospholipid cycles become significant at older ages or after an AD diagnosis.

Evidence for higher ATP utilization in midlife women is also in line with PET imaging reports of higher cerebral metabolic activity in women as compared to men across the adult lifespan^44,57^. FDG- and H_2_O-PET studies have shown that, as the brain ages, its resting metabolism gradually shifts away from a combination of nonoxidative and oxidative use of glucose to predominantly OXPHOS, an effect that seems more pronounced in women^57^ and that correlates with Aβ plaque distribution^58^. Our data add to these findings by providing novel evidence for higher brain ATP re-synthesis in women, which may reflect nonoxidative metabolism (e.g., aerobic glycolysis) being progressively replaced by OXPHOS^57^.

We did not observe sex differences in Pi/PCr (e.g. energy demand) or Pi/ATP (e.g. ATP hydrolysis^37^) except for lower Pi/ATP in PCC of women relative to men, which did not survive correction for multiple comparisons. The only other study that investigated sex differences in Pi/ATP showed lower ratios in temporal cortex, and higher ratios in parietal cortex of women as compared to men across the adult lifespan^36^. While the previous study did not examine PCC, these results provide preliminary evidence for emergence of sex differences in ATP hydrolysis during normal aging. The previous study also reported lower cortical energy demand in women^36^, whereas we found no sex differences in this metabolite ratio. Given the greater variability of Pi measures, resulting in greater standard deviations, it is possible that we were underpowered to detect significant effects. As some studies report changes in Pi/ATP or PCr/Pi in AD patients^30,45^, it is also possible that sex differences in these metabolite ratios become more significant at older ages or in presence of neurodegenerative disease. On the other hand, sex differences in ATP utilization relative to PCr are consistent across studies, supporting inclusion of this biomarker of mitochondrial function in studies of preclinical AD.

## Strengths and limitations

This study has several strengths. To our knowledge, this is the first ^31^P-MRS study to investigate the effects of sex and menopause status in a large group of well characterized, cognitively normal middle-aged individuals at risk for AD. All participants had brain MRI and ^31^P-MRS scans, clinical and cognitive exams, laboratory tests, APOE-4 status, and menopausal assessments. We acquired multi-slice 2D-CSI ^31^P-MRS scans that cover the whole brain, thus mapping multiple voxels over a whole grid instead of large single voxels, which enabled us to simultaneously assess HEP and phospholipid metabolites in a panel of AD-vulnerable brain regions. Results were significant after a stringent correction for multiple comparisons, which limits the potential for false positives, and after multi-variable correction for age, APOE-4 status, midlife health indicators, menopause-related factors, and intracranial volume.

All our participants were cognitively intact, and cognitive performance did not differ by sex or menopause status. The association of ATP/PCr and cognitive measures was also not significant. While we focused on cognitive tests known to be sensitive to estrogen changes, it is possible that different tests might yield different results. As our sample was highly educated, results may not apply to individuals of diverse educational status. Nonetheless, these results indicate that changes in mitochondrial function may be a more sensitive tool in detecting early signs of AD than neuropsychological testing currently available.

Evaluation of metabolite peak areas using XSOS is operator-dependent and may introduce subjective errors due to phase distortions and baseline roll although the same 1st-order phase shift was applied to all participants. While this might be a source of methodological bias, our technique is well validated^59,60^ and all fittings were performed by the same MRI physicist with over 20 years of experience in processing MRS data (JPD), which eliminates concerns around inter-rater variability. As pointed out by others, ^31^P-MRS measurements are affected by many acquisition characteristics such as transmit and receive field variation, SNR, and partial volume averaging^36,61^. All our participants were scanned with the same dual tuned ^31^P/^1^H birdcage head coil with identical transmit and receive gains. Our analysis focused on metabolite ratios and not absolute concentrations, as the latter are more sensitive to such factors. The fact that absolute quantification of metabolite levels is prone to error, and only relative metabolite levels can be computed, limits interpretability of ^31^P-MRS data. Future magnetization transfer experiments to determine the CK and ATPase reaction rates with measurements of absolute concentrations in relation to AD risk are warranted. Although our middle-aged participants are unlikely to exhibit substantial brain atrophy, partial-volume correction of ^31^P-MRS data is unattainable, which is a limitation in comparing men and women, especially in older participants.

Another limitation of ^31^P-MRS lies in its inherently low resolution given the low concentration (~ 1-10 mM) of metabolites. To address this limitation, our protocol involves acquisition of a high resolution T2-Weighted MRI image at exactly the same location as our ^31^P-MRS slices, performed immediately prior to the MRS scan. We then are able to accurately co-register this hi-resolution T2 with the 3D T1-Weighted image for analysis. The co-registration of PET with 3DT1 MRI was performed using state-of-the-art coregistration techniques^62^ according to published protocols^9–14^, and individually verified by overlaying each PET on the corresponding MRI after processing to ensure no discrepancies occurred in the registration process.

More work is also needed to determine what range of ATP utilization represents a biologically meaningful change. From a clinical perspective, the answer is likely multifactorial and depend upon individual metabolic utilization, sex, age, and clinical diagnosis, among other factors. From a statistical perspective, regional inter- and intra-day coefficient of variations are generally below 10%^63,64^. In our study, women exhibited 17% higher ATP/PCr levels in frontal cortex as compared to men (the difference was 18% for post-menopausal women), which we interpret as a statistically and biologically meaningful difference.

Currently, there are no ^31^P-MRS studies that tested for associations with AD biomarkers such as Aβ plaques. We caution that, although presence of Aβ plaques is a strong risk factor for AD, over 20% of healthy elderly exhibit cerebral Aβ burden in absence of dementia^65^, and about 6% of middle-age people also test positive^65^. In keeping with these estimates, 8% of women in our cohort who underwent Aβ PET imaging exhibited high brain Aβ burden according to pre-existing cut-offs derived from AD patients^66,67^. While some of these women with emerging Aβ pathology may eventually develop AD, it is also possible that Aβ deposition might reflect accelerated biological aging triggered by hormonal decline^68^ and related changes in mitochondrial function. We caution interpretation of our results as our participants are younger compared to those with a clinical diagnosis of AD, free of cognitive impairment, and in good general health. We also acknowledge that our participants’ relatively young age necessarily resulted in largely low levels of Aβ, thus conservatively reducing statistical power to detect associations with bioenergetic aging factors. Nonetheless, many studies demonstrate that examination of PiB signal as a continuous measure is sensitive enough to detect abnormalities among asymptomatic middle-aged at-risk individuals with apparently minimal pathology^69,70^. Present results of significant female-specific increases in ATP utilization in absence of equivalent alterations in Aβ distribution suggest that ATP changes may precede significant Aβ deposition in women. In this case, associations between ATP/PCr and fibrillar Aβ load may then become evident at older ages or with established disease. Alternatively, changes in ATP use may be secondary to the toxic effects of soluble Aβ, which is not detectable with PiB-PET. Mitochondria are intracellular targets of soluble Aβ oligomers, which cause overproduction of reactive oxygen species (ROS), disrupt intracellular calcium homeostasis, and trigger neuronal apoptosis prior to fibrilization^20^. Given the slow progressive nature of AD pathology, and the need to conduct early detection investigations during midlife years, 10–20 years of follow-ups may be needed to provide definitive conclusions as to how sex differences in brain bioenergetic factors might impact Aβ accumulation trajectories. We offer that our results provide preliminary metabolic and anatomical targets for future longitudinal studies combining ^31^P-MRS measures with AD-specific biomarkers, such as amyloid-PET and blood-based Aβ biomarkers.

Finally, present results were found in healthy, well-educated, mostly white individuals of generally middle/high socioeconomic status, which limits the generalizability of our findings.

## Conclusions

The present ^31^P-MRS study of middle-aged individuals at risk for AD indicates sex differences in brain metabolic function of AD-vulnerable regions during midlife endocrine aging. Such changes may increase susceptibility to AD in women and provide a window of opportunity for precision medicine based preventative strategies.

## Methods

### Participants and data

This is a natural history, non-interventional study of healthy, cognitively normal men and women ages 40–65 years, carrying risk factors for late-onset AD such as an AD family history and/or APOE-4 genotype. Participants were recruited at the Weill Cornell Medicine (WCM) Alzheimer’s Prevention Program between 2018 and 2022 by self-referral, flyers, and word of mouth.

Our inclusion and exclusion criteria have been previously described^9–14^. Briefly, all participants had Montreal Cognitive Assessment (MoCA) score ≥ 26 and normal cognitive test performance by age and education^9–14^. Exclusion criteria included medical conditions that may affect brain structure or function (e.g., stroke, any neurodegenerative diseases, major psychiatric disorders, hydrocephalus, demyelinating disorders such as Multiple Sclerosis, intracranial mass, and infarcts on MRI), use of psychoactive medications, and contraindications to MRI or PET imaging. All received medical, neurological, laboratory, cognitive and MRI exams, including volumetric MRI and ^31^P-MRS within 6 months of each other. A subset of 97 participants, including 78 (80%) women and 19 (20%) men, also received ^11^C-PiB scans within approximately 3 months of the MRI scans.

The patients’ sex was determined by self-report. APOE-4 genotype was determined using standard qPCR procedures^9–14^. Participants carrying one or two copies of the APOE-4 allele were grouped together as APOE-4 carriers, and compared to non-carriers.

### Standard protocol approvals, registrations, and patient consents

All methods were carried out in accordance with relevant guidelines and regulations. All experimental protocols were approved by the WMC Institutional Review Board. Written informed consent was obtained from all participants.

### Menopause status

Determination of menopausal status was elicited using standardized questionnaires based on the Stages of Reproductive Aging Workshop (STRAW) criteria^71^. Participants were classified as pre-menopausal (regular cyclers), peri-menopausal (irregular cyclers with an interval of amenorrhea ≥ 60 days or ≥ 2 skipped cycles), or post-menopausal (absence of menstrual cycle for 12 or more months) using hormone laboratory assessments as supportive criteria^14^. Menopause type (spontaneous vs. induced) was assessed through a series of questions related to gynecological surgery (hysterectomy and/or oophorectomy) before menopause^14^. Use of menopause hormone therapy (HT) was elicited using standardized questionnaires^14^.

### Cognitive measures

We used the delayed recall from Rey Auditory Verbal Learning Test (RAVLT) and delayed recall from Wechsler Memory Scale logical memory, the FAS test, animal naming, and Trail Making Test B (TMT-B total score), all tests with known sensitivity to estrogen levels^12,72^, to derive composite global cognition and memory scores (see “Statistical analysis”).

### Image acquisition and analysis

#### Magnetic resonance imaging and spectroscopy

All participants received a 3D volumetric T_1_-weighted MRI scan on a 3.0 T GE MR 750 Discovery scanner (General Electric, Waukesha, WI) [BRAVO; 1 × 1 × 1 mm resolution, 8.2 ms repetition time (TR), 3.2 ms echo time (TE), 12° flip angle, 25.6 cm field of view (FOV), 256 × 256 matrix with ARC acceleration] using a 32-channel head coil. The ^31^P-MRS scan was acquired on the same scanner as the MRI, typically on the same day, using a dual tuned ^31^P/^1^H quadrature head coil (Ralph Hashoian; Clinical MR Solutions, Brookfield, WI). Prior to MRS scanning, shimming was performed using a ^1^H single voxel technique placed over the entire brain avoiding the air-tissue interfaces. Multiple 2D slices were acquired resulting in an 8 × 8x8 grid with a 24 cm FOV. Spectroscopic imaging parameters included 2048 points, 5000 Hz sweep width, 2000 ms TR, 2 averages, 55° flip angle at 51.3 MHz in the sagittal plane. After ^31^P-MRS was complete, an 8 slice sagittal T_1_-Fluid Attenuated Inversion Recovery sequence [FLAIR; 2200 ms TR, 12 ms TE, 780 ms inversion time (TI), 24 cm FOV, 0.94 × 0.94 mm] was acquired with a 5 mm slice thickness at exactly the same position as the center of each ^31^P MRS slice for reference.

#### Amyloid-β positron emission tomography

^11^C-Pittsburgh Compound B (PIB) PET scans were acquired with on a Siemens BioGraph mCT PET/CT scanner operating in 3D mode, following standardized procedures^10,11,13^. All scans were performed in the awake, eyes-closed state, summing the activity 60–90 min post-injection of 15 mCi of ^11^C-PiB. All images were corrected for attenuation, scatter and decay. Images were reconstructed using PSF and TOF techniques to a resolution of 0.8 × 0.8 × 3.0 mm with 74 slices.

#### Multiparametric mapping

MRS data were processed using XSOS^59,60^ (Dikoma Shungu/Xiangling Mao; Weill Cornell Medicine) written in IDL (Excelis Visual, Boulder, CO). Raw data were loaded into the program and processed using Hamming and Fermi k-space filters, a 7.5 mm center voxel shift, 20 Hz exponential filtering and zero-filling in time, x and y-domains prior to 3D Fast Fourier Transformation. A fixed first order phase of 4200° was applied to all spectra and data were automatically phased in zero order. The PCr peak was set at 0.0 ppm and the central spectrum set as a reference, and susceptibility corrections performed throughout the data set. Baseline correction was applied to all other voxels in the CSI data set by an experienced analyst (JPD).

Peak area integration was performed around each of the seven well-resolved resonance peaks identified in Supplementary Information Fig. 1: inorganic phosphate (Pi), phospho-creatine (PCr), ATP (α-ATP, β-ATP and γ-ATP moieties), phosphodiesters (PDE), and phosphomonoesters (PME). This creates a 16 × 16 image of voxels 1.5 × 1.5 × 3.0 cm with the signal intensity in each voxel corresponding to the peak area of the ^31^P metabolite. The integral of each metabolite resonance was calculated and expressed as a percent area of the total phosphorous signal in the corresponding spectrum. The ratios ATP/PCr, Pi/PCr, Pi/ATP, and PME/PDE were then computed as this allows for normalization of the data. ATP/PCr is a measure of energy utilization^12^, Pi/PCr is a measure of energy demand^36^, Pi/ATP is a measure of ATP transferase^37^, and PME/PDE is a measure of phospholipid turnover^38^.

The 3D T_1_-Weighted BRAVO MRI scan was automatically processed in FreeSurfer 6.0 running under the Centos 7 Linux environment and Statistical Parametric Mapping (SPM12)^62^ (http://www.fil.ion.ucl.ac.uk/spm/) implemented in Matlab 2021 (MathWorks; Natick, MA) using automated piplines^9–14^. For each participant, the central 4 slices of the MRS scan were co-registered to the T_1_ MRI sequence by using the 8-slice concordant image set acquired at the time of MRS. The parametric metabolite MRS maps and corresponding PiB-PET scan were co-registered with the skull stripped MRI using the Normalized Mutual Information routine of SPM8^62^. Volumetric MRI and PiB-PET scans were resampled to a 256 × 256 × 256 matrix array whereas the parametric metabolite MRS maps were resized to 256 × 256 images but not interpolated beyond the original 16 × 16 × 8 matrix.

The co-registered MRI, MRS maps, and PET images were quantified using the subcortical gray and white matter segmentation tools implemented in FreeSurfer 6.0 and Desikan-Killiany Atlas-based regions of interest (ROIs)^73,74^ applied to the aligned MRI. ROIs were overlaid onto each of the co-registered MRS and PET scans for regional sampling. We focused on brain regions with known metabolic vulnerability to metabolic aging and AD, including: frontal cortex (middle and superior frontal gyrus); PCC (posterior cingulate gyrus and precuneus); temporal cortex (inferior, middle and superior temporal gyrus); fusiform gyrus; and medial temporal lobe (hippocampus, amygdala, entorhinal and parahippocampal gyrus)^47,48^. We also obtained total intracranial volume for normalization purposes.

For quantification of PiB-derived Aβ burden, we created an AD mask as the average of ROIs preferentially affected by Aβ deposition, including: inferior parietal lobule; superior, inferior and middle temporal gyri; superior, middle and medial frontal gyri; PCC and precuneus^66,67^. PiB uptake in AD-mask and individual ROIs was normalized to cerebellar GM uptake, also via FreeSurfer, to obtain standardized uptake value ratios (SUVR)^9–14^. PiB uptake was examined as a continuous measure. For descriptive purposes, participants were also classified as exhibiting high or low levels of cerebral amyloidosis based on a published cut-off of AD-mask SUVR > 1.42, as derived from studies of AD patients^66,67^.

### Covariates

All analyses were adjusted by age (years), APOE-4 status (carrier vs. non-carrier) and total intracranial volume (cc). Cognitive measures were further adjusted by education (years). For exposures showing significant associations with outcome measures, we examined additional confounders including (i) midlife health indicators: smoking (present vs. past vs. never smoker), hypertension (systolic blood pressure ≥ 140 mmHg or diastolic blood pressure ≥ 90 mmHg and/or use of anti-hypertensive medications), hypercholesterolemia (plasma cholesterol ≥ 240 mg/dL), and hyperinsulinemia (HOMA-IR > 1.8); and (ii) menopause-related factors: type of menopause (spontaneous vs. surgical) and history of HT use (user vs. never-user).

### Statistical analysis

Analyses were performed in R version 4.2.0 and SPSS v.25. Clinical measures were examined with general linear models or chi-squared tests as appropriate. Cohort characteristics are described using mean (standard deviation) and n, percentage (%), stratified by menopause exposure group.

All cognitive outcomes are continuous and were scaled to standard deviations and centered at 0. The composite memory score was obtained by z-scoring each delayed memory test (RAVLT, logical memory) and averaging across measures. TMT-B scores were inverted so that positive Z scores reflected better performance, prior to averaging. Global cognition scores were then obtained by z-scoring the remaining tests and averaging across these scores and the composite memory score. Regression models for each cognitive outcome were trained containing first a two-level exposure variable (levels: men, women), and secondly a four-level exposure variable (levels: male, pre-menopause, peri-menopause, post-menopause), adjusting by the confounders listed above. Results were considered significant at p < 0.05, after Bonferroni correction for multiple comparisons.

All brain imaging outcomes are continuous and were scaled to standard deviations and centered at 0. While several publications report PCr/Pi ratios, due to the small magnitude and high variability (standard deviation) in the Pi measures, we obtained Pi to PCr ratios in order to obtain more stable estimates and confidence bounds for multivariable linear regression modeling. ATP/PCr ratios were also used for consistency for regression modeling. However, PCr/Pi and PCr/ATP measures are presented in Supplementary Information Tables 1 and 2 to enable comparisons with previous publications.

Multivariable linear regression models for each metabolite outcome were trained to consider the effect of a two-level exposure variable (levels: men, women) across all brain regions. Regression models were constructed to obtain global P values to test for multivariate outcomes across all brain regions for each metabolite. All analyses were adjusted by age, APOE-4 status and total intracranial volume. Models showing significant effects of exposures after these adjustments were re-evaluated including the additional confounders listed above. Pairwise comparisons between each exposure level were made after Bonferroni multiple comparisons adjustment at p < 0.05. Bonferroni-corrected p values are provided for all results. For models showing significant main effects, regional differences between groups were explored using forest plots where estimated effect size in excess of the test–retest variability in ^31^P-MRS measures (e.g. ≥ 10% difference^63,64^) were considered clinically and statistically meaningful.

Metabolite outcomes showing significant sex effects (e.g. ATP/PCr) were then examined with multivariate regression models trained to consider the effect of a four-level exposure variable on ATP/PCr across all brain regions (levels: male, pre-menopause, peri-menopause, post-menopause) adjusting by confounders, and after Bonferroni multiple comparisons adjustment, at p < 0.05. Regression models were constructed to obtain global P values to test for multivariate differences in any menopause status levels for each given outcome variable. This approach yields the significance of the 4-level variable as a whole in the model. Regional group differences were then explored using forest plots as described above.

Finally, we used linear regressions to test for associations of regional metabolite levels showing significant sex effects with PiB SUVR in AD mask and in the region showing the largest effect size (e.g. frontal cortex), adjusting for the confounders. A smoothing spline was used to estimate an overall fit at p < 0.05.

## Supplementary Information

Supplementary Figure 1.

Supplementary Tables.

## Data Availability

The datasets analyzed during the current study may be made available from the corresponding author on reasonable request.
