# Supplementary material for: Sex and menopause impact 31P-Magnetic Resonance Spectroscopy brain mitochondrial function in association with 11C-PiB PET amyloid-beta load
Source: Sci Rep. 2022 Dec 21;12:22087. doi: 10.1038/s41598-022-26573-5 (PMC9772209; doi:10.1038/s41598-022-26573-5)
Supplement: Supplementary file 1 — Supplementary Figure 1. [file 41598_2022_26573_MOESM1_ESM.docx]

#### e-Figure 1. ^31^P-MRS spectra of two representative participants


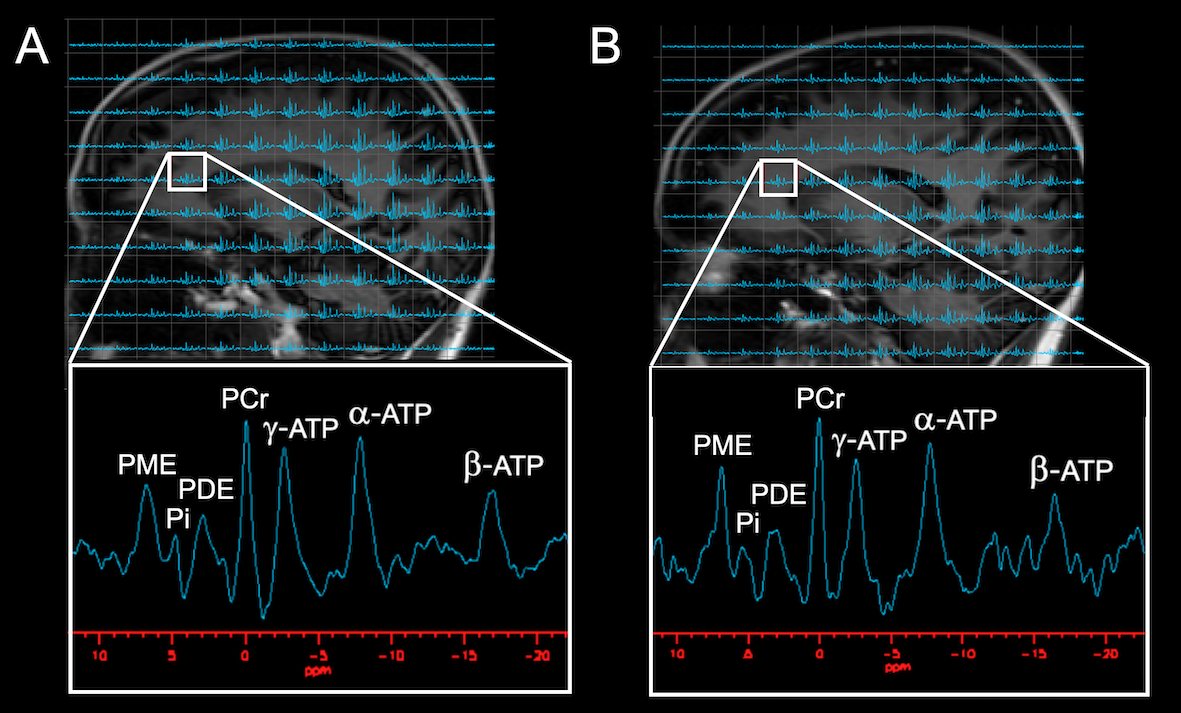


Seven well-resolved resonance peaks were identified, including the HEP molecules phosphocreatine (PCr), adenosine triphosphate (α-ATP, β-ATP and γ-ATP), and inorganic phosphate (Pi); and phospholipid metabolites phosphomonoesters (PME) and phosphodiesters (PDE). P_i_ and the phospholipids are located to the left of PCr. Resonant peaks from the three phosphate groups of ATP (γ-, α-, and β-ATP from left to right) are located to the right of PCr.

A spectrum from the frontal lobe is shown in (A) a 55 year-old post-menopausal woman exhibiting decreased PCr/ATP and (B) a pre-menopausal woman exhibiting increased PCr/ATP.
