# Supplementary material for: Sex and menopause impact 31P-Magnetic Resonance Spectroscopy brain mitochondrial function in association with 11C-PiB PET amyloid-beta load
Source: Sci Rep. 2022 Dec 21;12:22087. doi: 10.1038/s41598-022-26573-5 (PMC9772209; doi:10.1038/s41598-022-26573-5)
Supplement: Supplementary file 2 — Supplementary Tables. [file 41598_2022_26573_MOESM2_ESM.docx]

# Supplementary Information Table 1. Sex differences in PCr/ATP and PCr/Pi ratios

| Metabolite | Region | Group | Mean | Std. Error | 95% C.I. | |
| --- | --- | --- | --- | --- | --- | --- |
| PCr/ATP | Frontal | Women | 2.509 | 0.038 | 2.434 | 2.583 |
|  |  | Men | 2.916 | 0.082 | 2.755 | 3.078 |
|  | Temporal | Women | 3.126 | 0.032 | 3.063 | 3.190 |
|  |  | Men | 3.286 | 0.070 | 3.148 | 3.423 |
|  | PCC / Precuneus | Women | 2.794 | 0.037 | 2.720 | 2.867 |
|  |  | Men | 3.011 | 0.081 | 2.852 | 3.170 |
|  | Medial temporal | Women | 4.178 | 0.042 | 4.094 | 4.261 |
|  |  | Men | 4.397 | 0.091 | 4.217 | 4.577 |
|  | Fusiform | Women | 1.099 | 0.011 | 1.078 | 1.121 |
|  |  | Men | 1.149 | 0.023 | 1.103 | 1.195 |
| PCr/Pi | Frontal | Women | 11.056 | 0.336 | 10.394 | 11.718 |
|  |  | Men | 14.032 | 0.726 | 12.601 | 15.463 |
|  | Temporal | Women | 18.415 | 0.391 | 17.643 | 19.186 |
|  |  | Men | 20.521 | 0.846 | 18.854 | 22.189 |
|  | PCC | Women | 12.801 | 0.330 | 12.151 | 13.450 |
|  |  | Men | 13.756 | 0.712 | 12.351 | 15.160 |
|  | Medial temporal | Women | 24.144 | 0.761 | 22.643 | 25.645 |
|  |  | Men | 28.601 | 1.646 | 25.357 | 31.845 |
|  | Fusiform | Women | 5.930 | 0.133 | 5.667 | 6.192 |
|  |  | Men | 6.296 | 0.288 | 5.729 | 6.864 |

Multi-variable adjusted, unstandardized mean (SE) and 95% confidence intervals (CI). While in the main analysis of sex effects we examined ATP/PCr and Pi/PCr ratios, PCr/ATP and PCr/Pi measures are presented in this table to enable comparison with previous publications.

Abbreviations: ATP, total adenosine triphosphate; PCC, posterior cingulate cortex and precuneus; PCr, phosphocreatine; Pi, inorganic phosphate.

# Supplementary Information Table 2. Menopause effects on PCr/ATP levels

| Region | Group | Mean | Std. Error | 95% C.I. | |
| --- | --- | --- | --- | --- | --- |
| Frontal | Pre-menopause | 2.541 | 0.089 | 2.367 | 2.716 |
|  | Peri-menopause | 2.504 | 0.063 | 2.380 | 2.628 |
|  | Post-menopause | 2.493 | 0.067 | 2.362 | 2.625 |
|  | Men | 2.917 | 0.082 | 2.755 | 3.080 |
| Temporal | Pre-menopause | 3.186 | 0.075 | 2.938 | 3.233 |
|  | Peri-menopause | 3.191 | 0.053 | 3.086 | 3.295 |
|  | Post-menopause | 3.095 | 0.056 | 2.984 | 3.206 |
|  | Men | 3.283 | 0.070 | 3.145 | 3.420 |
| PCC | Pre-menopause | 2.762 | 0.087 | 2.591 | 2.933 |
|  | Peri-menopause | 2.864 | 0.061 | 2.743 | 2.985 |
|  | Post-menopause | 2.753 | 0.065 | 2.624 | 2.881 |
|  | Men | 3.008 | 0.081 | 2.849 | 3.167 |
| Medial temporal | Pre-menopause | 4.200 | 0.098 | 3.906 | 4.293 |
|  | Peri-menopause | 4.252 | 0.070 | 4.115 | 4.389 |
|  | Post-menopause | 4.158 | 0.074 | 4.013 | 4.304 |
|  | Men | 4.393 | 0.091 | 4.213 | 4.573 |
| Fusiform | Pre-menopause | 1.183 | 0.025 | 1.033 | 1.132 |
|  | Peri-menopause | 1.121 | 0.018 | 1.086 | 1.156 |
|  | Post-menopause | 1.090 | 0.019 | 1.053 | 1.127 |
|  | Men | 1.148 | 0.023 | 1.102 | 1.194 |

Multi-variable adjusted, unstandardized mean (SE) and 95% confidence intervals (CI). While in the main analysis of menopause status we examined ATP/PCr ratios, PCr/ATP measures are presented in this table to enable comparison with previous publications.

Abbreviations: ATP, total adenosine triphosphate; PCC, posterior cingulate cortex and precuneus; PCr, phosphocreatine.
